# Supplementary material for: Acute exercise rewires the proteomic landscape of human immune cells
Source: Nat Commun. 2026 Jan 2;17:130. doi: 10.1038/s41467-025-68101-9 (PMC12775541; doi:10.1038/s41467-025-68101-9)
Supplement: Supplementary file 1 — Supplementary Information [file 41467_2025_68101_MOESM1_ESM.pdf]

Acute exercise rewires the proteomic landscape of human immune cells

Supplementary information

David Walzik,[1,*] Niklas Joisten,[1,*] Alan J Metcalfe,[2,3] Sebastian Proschinger,[1] Alexander Schenk,[1] Charlotte Wenzel,[1] Alessa L. Henneberg,[4] Martin Schneider,[5] Silvia Calderazzo,[6] Andreas Groll,[7] Carsten Watzl,[8] Christiane A. Opitz,[4] Dominic Helm,[5] Philipp Zimmer[1,#]

[1]Sports Medicine Research Group, Institute for Sport and Sport Science, TU Dortmund University, Otto-Hahn-Strasse 3, 44227 Dortmund, Germany

[2]Department of Molecular and Cellular Sports Medicine, Institute of Cardiovascular Research and Sports Medicine, German Sport University Cologne, Am Sportpark Müngersdorf 6, 50933 Cologne, Germany

[3]Chest Unit, Centre for Human and Applied Physiological Sciences (CHAPS), Denmark Hill Campus, King's College Hospital, King's College London, London, United Kingdom

[4]German Cancer Research Center (DKFZ), Heidelberg, Division of Metabolic Crosstalk in Cancer and the German Cancer Consortium (DKTK), DKFZ Core Center Heidelberg, Im Neuenheimer Feld 280, 69120 Heidelberg, Germany

[5]Proteomics Core Facility, German Cancer Research Center (DKFZ), Im Neuenheimer Feld 280, 69120 Heidelberg, Germany

[6]Division of Biostatistics, German Cancer Research Center (DKFZ), Im Neuenheimer Feld 280, 69120 Heidelberg, Germany

[7]Department of Statistics, TU Dortmund University, Vogelpothsweg 87, 44227 Dortmund, Germany

[8]Leibniz Research Center for Working Environment and Human Factors at TU Dortmund (IfADo), Ardeystrasse 67, 44139 Dortmund, Germany

*These authors contributed equally

[#]Correspondence: philipp.zimmer@tu-dortmund.de (P.Z.)

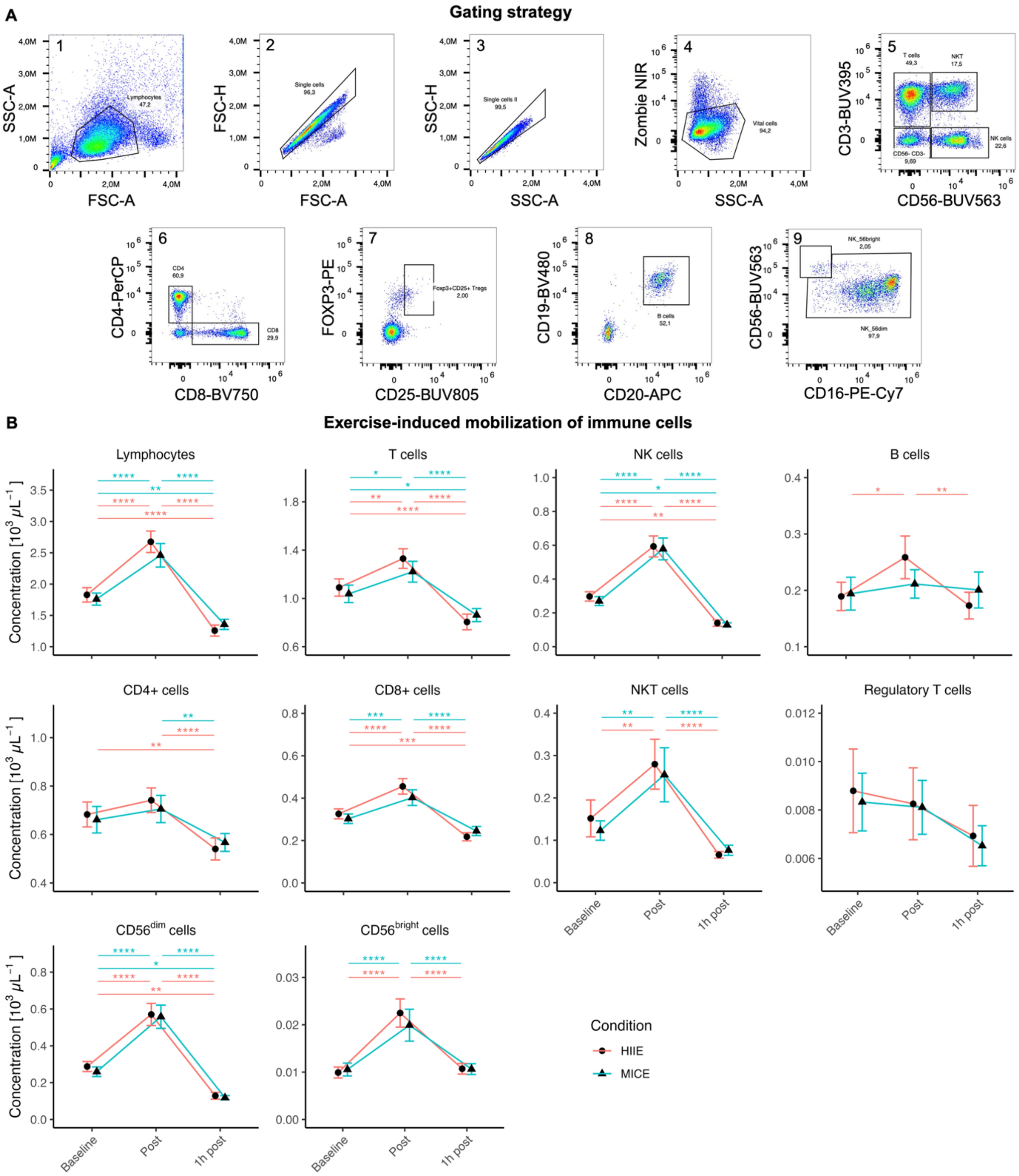

Figure S1 Immune cell mobilization is independent of exercise intensity

(A) Gating strategy applied to PBMCs. Gated immune cell populations included vital lymphocytes, T cells ($CD3^+CD56^-$) either as CD8+ T cells ($CD3^+CD56^-CD8^+CD4^-$) or CD4+ T cells ($CD3^+CD56^-CD8^-CD4^+$), natural killer (NK) T cells ($CD3^+CD56^+$), regulatory T cells ($CD3^+CD56^-CD8^-CD4^+CD25^+Foxp3^+$), NK cells ($CD3^-CD56^+$) either as NK^{bright} ($CD3^-CD56^+CD56^{bright}CD16^-$) or NK^{dim} ($CD3^-CD56^+CD56^{dim}CD16^+$), and B cells ($CD3^-CD56^-CD19^+CD20^+$).

(B) Mobilization kinetics of total lymphocytes and lymphocyte subsets in response to high-intensity interval exercise (HIIE) and moderate-intensity continuous exercise (MICE). Linear mixed models with subsequent analyses of variance and Bonferroni-corrected pairwise comparisons were applied. Data are represented as mean ± SEM (N = 22). Time effects: **** $p < 0.0001$, *** $p < 0.001$, ** $p < 0.01$, * $p < 0.05$.

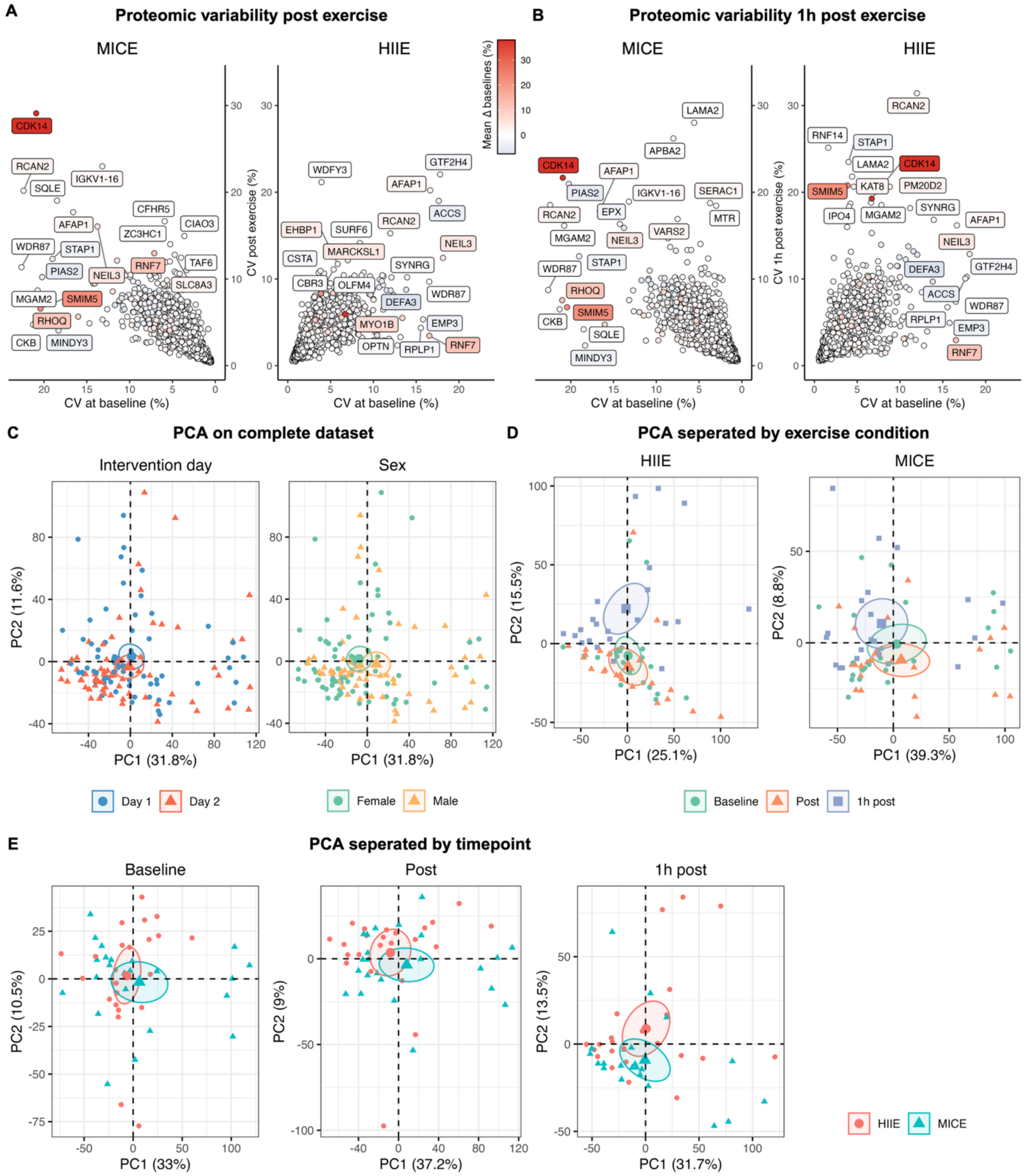

Figure S2 Proteomic variability and principal component analyses (PCA) separated by exercise condition and measurement timepoint
(A) Proteomic variability immediately after HIIE and MICE.
(B) Proteomic variability 1h after HIIE and MICE.
(C) PCA on complete dataset to evaluate the impact of intervention day and sex.
(D) PCA separated by exercise condition to evaluate the impact of measurement timepoint.
(E) PCA separated by measurement timepoint to evaluate the impact of exercise condition.

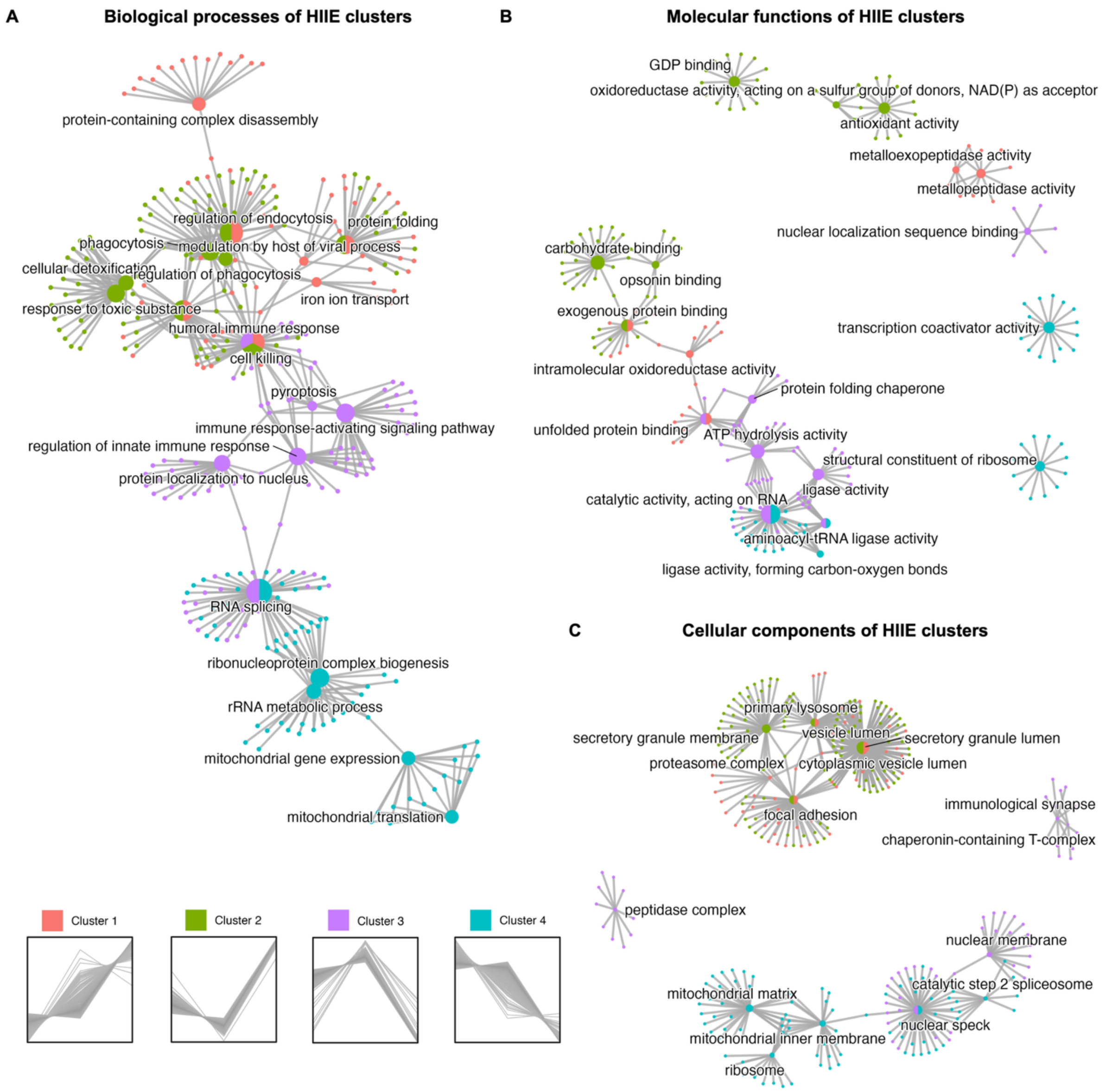

Figure S3 Biological theme comparison suggests shared and unique GO terms across protein clusters in HIIE
(A) Comparison of biological processes in clusters 1 – 4.
(B) Comparison of molecular functions in clusters 1 – 4.
(C) Comparison of cellular components in clusters 1 – 4.

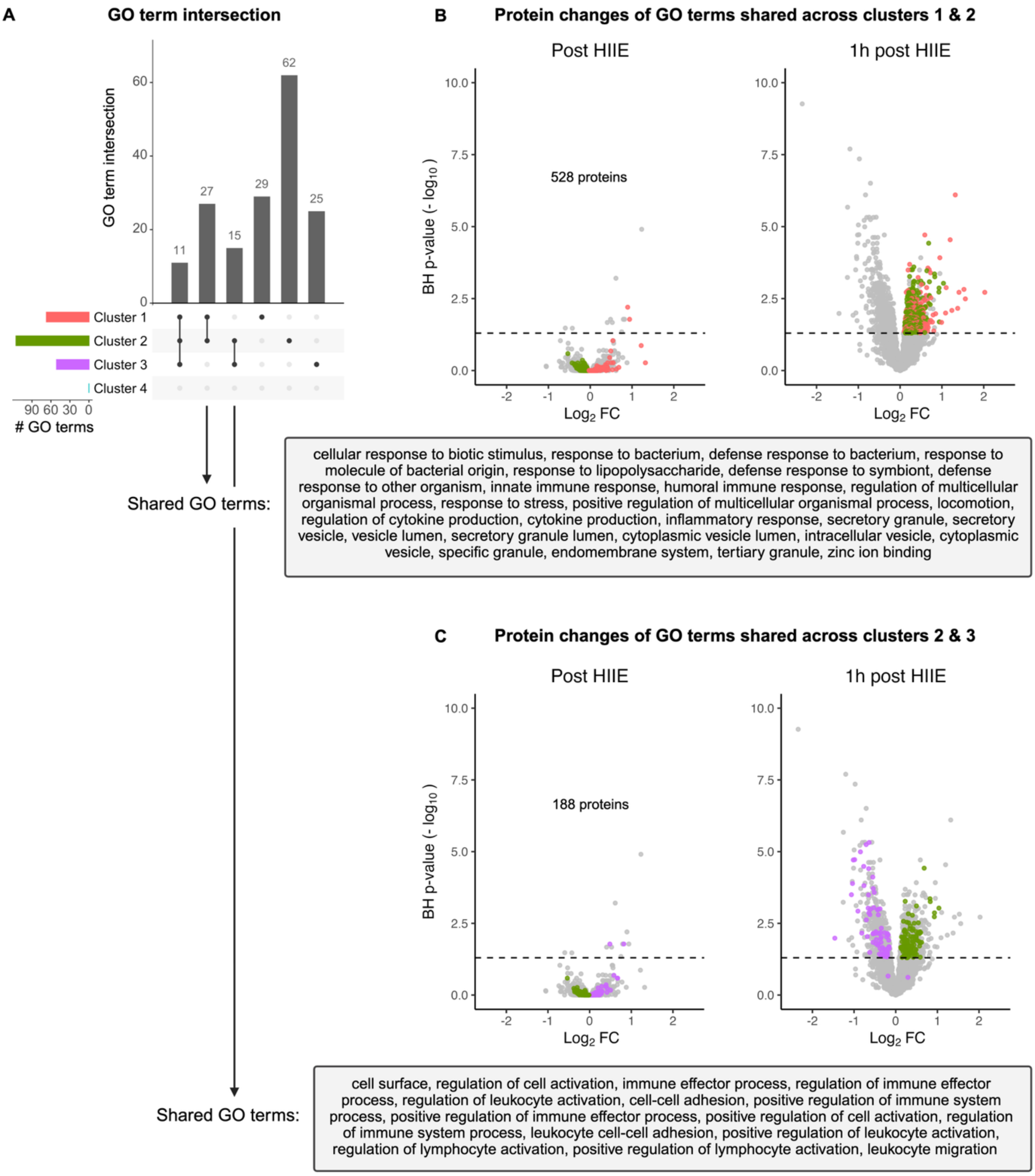

Figure S4 GO terms overlap between clusters 1 & 2 and 2 & 3, respectively
(A) Overview of shared and unique GO terms across all clusters.
(B) GO terms shared between clusters 1 and 2.
(C) GO terms shared between clusters 2 and 3.

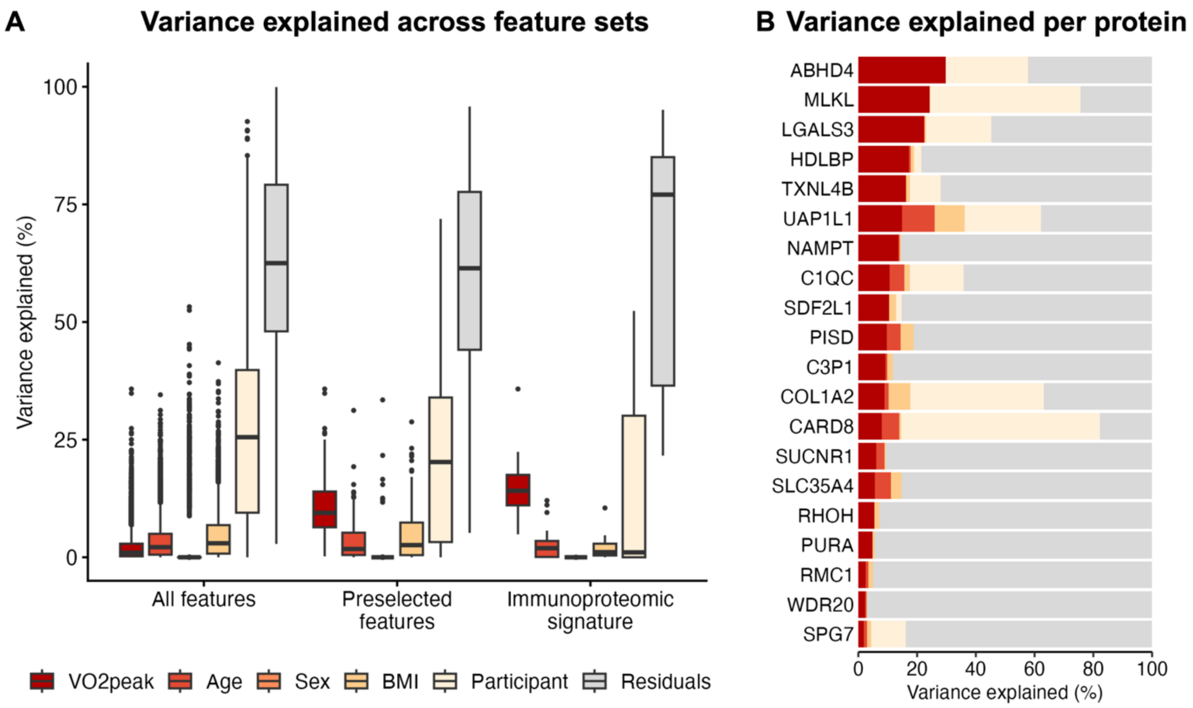

Figure S5 Full results of variance partitioning and correlation between $\dot{V}O_{2peak}$ and NAMPT protein levels obtained by untargeted proteomics
(A) Variance explained by participant characteristics and residual variance across different feature sets (all features, preselected features, immunoproteomic signature).
(B) Variance explained by participant characteristics and residual variance for each protein of the immunoproteomic signature.
