# Supplementary material for: Acute exercise rewires the proteomic landscape of human immune cells
Source: Nat Commun. 2026 Jan 2;17:130. doi: 10.1038/s41467-025-68101-9 (PMC12775541; doi:10.1038/s41467-025-68101-9)
Supplement: Supplementary file 2 — Description of Additional Supplementary Files [file 41467_2025_68101_MOESM2_ESM.pdf]

Description of Additional Supplementary Files

Supplementary Data S1. Allocation of raw data files to study participants.

Supplementary Data S2. Immune cell counts from flow cytometry analysis and FlowSOM results.

Supplementary Data S3. Detailed statistical results from analysis of exercise-induced changes in immune cell counts.

Supplementary Data S4. Lymphocyte proportions from flow cytometry and detailed statistical results from analysis of exercise-induced changes.

Supplementary Data S5. Detailed statistical results from analysis of exercise-induced changes in protein abundance of PBMCs.

Supplementary Data S6. Detailed results of Gene ontology over-representation analysis.

Supplementary Data S7. Membership values and fold changes from baseline of all proteins used for fuzzy c-means clustering.

Supplementary Data S8. Detailed results of biological theme comparison.

Supplementary Data S9. Detailed results of all Gene ontology gene set enrichment analyses.

Supplementary Data S10. Detailed results of $\dot{V}O_{2peak}$ prediction.
