# Supplementary material for: Acute exercise rewires the proteomic landscape of human immune cells
Source: Nat Commun. 2026 Jan 2;17:130. doi: 10.1038/s41467-025-68101-9 (PMC12775541; doi:10.1038/s41467-025-68101-9)
Supplement: Supplementary file 13 — Reporting Summary [file 41467_2025_68101_MOESM13_ESM.pdf]

nature portfolio

Corresponding author(s): Philipp Zimmer

Last updated by author(s): Dec 15, 2025

Reporting Summary

Nature Portfolio wishes to improve the reproducibility of the work that we publish. This form provides structure for consistency and transparency in reporting. For further information on Nature Portfolio policies, see our Editorial Policies and the Editorial Policy Checklist.

Statistics

For all statistical analyses, confirm that the following items are present in the figure legend, table legend, main text, or Methods section.

n/a	Confirmed	
☐	☒	The exact sample size (*n*) for each experimental group/condition, given as a discrete number and unit of measurement
☐	☒	A statement on whether measurements were taken from distinct samples or whether the same sample was measured repeatedly
☐	☒	The statistical test(s) used AND whether they are one- or two-sided<br>*Only common tests should be described solely by name; describe more complex techniques in the Methods section.*
☐	☒	A description of all covariates tested
☐	☒	A description of any assumptions or corrections, such as tests of normality and adjustment for multiple comparisons
☐	☒	A full description of the statistical parameters including central tendency (e.g. means) or other basic estimates (e.g. regression coefficient) AND variation (e.g. standard deviation) or associated estimates of uncertainty (e.g. confidence intervals)
☐	☒	For null hypothesis testing, the test statistic (e.g. *F*, *t*, *r*) with confidence intervals, effect sizes, degrees of freedom and *P* value noted<br>*Give P values as exact values whenever suitable.*
☒	☐	For Bayesian analysis, information on the choice of priors and Markov chain Monte Carlo settings
☐	☒	For hierarchical and complex designs, identification of the appropriate level for tests and full reporting of outcomes
☐	☒	Estimates of effect sizes (e.g. Cohen's *d*, Pearson's *r*), indicating how they were calculated

Our web collection on statistics for biologists contains articles on many of the points above.

Software and code

Policy information about availability of computer code

Data collection	Cardiorespiratory fitness of participants was assessed using a Cortex Metalyzer 3B (CORTEX Biophysik GmbH, Leipzig, Germany) with the MetaSoft® Studio application software, which represents the basis of all CORTEX systems. Randomization of participants was performed using the software Randomization in Treatment Arms (RITA; Evidat, Lübeck, Germany). Flow cytometry analysis was performed using a Cytek® Aurora full spectrum flow cytometer with the associated SpectroFlo® software (Cytek Biosciences, California, USA). Analysis of total blood cell counts was performed from EDTA blood using a hematology analyzer with built-in software (SYSMEX XP-300, Norderstedt, Germany). Gating of flow cytometry results was performed using FlowJo™ 10.10.0 (BD Biosciences, NJ, USA). For the LC-MS/MS-based untargeted proteomics analysis an amount of 10 µg of protein per sample was digested (Trypsin) using an AssayMAP Bravo liquid handling system (Agilent technologies) running the autoSP3 protocol. The analysis was carried out on an Ultimate 3000 UPLC system (Thermo Fisher Scientific) directly connected to an Orbitrap Exploris 480 mass spectrometer. Instrument performance and suitability was monitored by regular (approx. one per 48 hours) injections of a standard sample and an in-house shiny application over the whole timeline of the experiment. Analysis of data independent acquisition RAW files was performed with Spectronaut (Biognosys, version 19.1.240724.62635) in directDIA+ (deep) library-free mode. Default settings were applied with the following adaptions. Within DIA Analysis under Identification the Precursor PEP Cutoff was set to 0.01, the Protein Qvalue Cutoff (Run) set to 0.01 and the Protein PEP Cutoff set to 0.01. In Quantification the Proteotypicity Filter was set to Only Protein Group Specific, the Protein LFQ Method was set to MaxLFQ and the quantification window was set to Not Synchronized (SN 17). The data was searched against the human proteome from Uniprot (human reference database with one protein sequence per gene, containing 20,597 unique entries from ninth of February 2024) and the contaminants FASTA from MaxQuant (246 unique entries from twenty-second of December 2022).
Data analysis	All statistical analyses were performed in R. If not otherwise noted, data wrangling was achieved using the dplyr and tidyr packages and visualization was subsequently achieved using the ggplot2 and ggpubr packages. No custom code was generated.<br>Unsupervised immune cell clustering was performed using the FlowAI plugin (v3.2.3), DownSample plugin (v3.3.1), and the FlowSOM plugin

(v4.1.0) in FlowJo™ 10.10.0. Exercise-induced alterations in immune cell counts were analyzed by applying linear mixed models to the flow cytometry results using the lmer function from the lme4 package. Results of the linear mixed models were then analyzed for time and time × condition interaction effects via analyses of variance (ANOVAs) with the built-in ANOVA function from R stats. In case of significant results, pairwise comparisons of measurement timepoints and/or exercise conditions were performed by applying the emmeans function from the emmeans package.
Principal component analysis (PCA) was carried out using the built-in prcomp function from R stats. All samples were plotted with the fviz_pca_ind function from the factoextra package.
A linear mixed model was fitted on the log2-transformed, normalized, and imputed protein intensities via the limma R package. Intra-individual correlation was estimated via the duplicateCorrelation function. A moderated t statistic was obtained for each contrast of interest via the eBayes function with estimated variance trend and robustification. The resulting p-values for each contrast were adjusted with the Benjamini-Hochberg procedure to control the false discovery rate and significance was declared at the adjusted 5 % two-sided level.
GO over-representation analysis was performed with the ClusterProfiler package. For HIIE and MICE, significantly altered proteins were compared with the entire dataset of quantified proteins applying Benjamini-Hochberg correction of p-values with a p-value cutoff of 0.05 and a q-value cutoff of 0.2.
Fuzzy c-means clustering was performed with the Mfuzz package. Data was standardized using the standardise function and the optimal number of clusters was determined by calculating the minimum centroid distance for a range of cluster numbers using the Dmin function. The optimal fuzzifier was identified with the mestamiate function.
Biological theme comparison was carried out using the compareCluster function from the ClusterProfiler package. Entrez gene identifiers of the proteins contained in the identified clusters were used as input with the function command set to “enrichGO”. Benjamini-Hochberg correction was applied to p-values with a cutoff of 0.05 and minimum gene set size was set to 10. The results were simplified via the simplify function using a cutoff of 0.7 and visualized separated by ontology with the cnetplot function from the enrichplot package.
Gene set enrichment analysis was performed using the gseGO function from the ClusterProfiler package. Entrez gene identifiers and fold changes from baseline of the proteins contained in the identified clusters were used as input with the minimum gene set size set to 10. In case fold changes were only positive or negative, the “scoreType” command was set to “pos” or “neg”, respectively. P-values were corrected using the Benjamini-Hochberg procedure with a p-value cutoff of 0.05. The underlying proteins mapping to each significant GO term were identified using the select function from the AnnotationDbi package. Shared and unique GO terms across the identified clusters were visualized with the UpSetR package.
To identify features with high association to VO2peak, we conducted a preselection in Python (v.3.9). We ran LASSO and ridge regression as well as a random forest as a non-linear, tree-based approach. A leave-one-out (LOO) cross-validation was performed in Python (v.3.9) to assess the predictive performance of these methods based on the preselected features. The 20 features with the largest absolute mean value from the ridge regression were selected to create a weighted, undirected network using Spearman’s rank correlations. The network was visualized in R (v.4.4.1) with the packages Hmisc (v.5.2.1) and igraph (v.2.1.1.).

For manuscripts utilizing custom algorithms or software that are central to the research but not yet described in published literature, software must be made available to editors and reviewers. We strongly encourage code deposition in a community repository (e.g. GitHub). See the Nature Portfolio guidelines for submitting code & software for further information.

Data

Policy information about availability of data

All manuscripts must include a data availability statement. This statement should provide the following information, where applicable:

- Accession codes, unique identifiers, or web links for publicly available datasets
- A description of any restrictions on data availability
- For clinical datasets or third party data, please ensure that the statement adheres to our policy

All data associated with this article can be explored via our interactive web application at https://sportsmedicine-dortmund.shinyapps.io/beat. Raw data files of all samples processed in the proteomics analysis are hosted on the PRoteomics IDEntifications Database (PRIDE; https://www.ebi.ac.uk/pride) under the following PRIDE-ID: PXD058573. Raw data files of all samples processed in the flow cytometry analysis are hosted on https://figshare.com under the following digital object identifier (DOI): 10.6084/m9.figshare.30543317. To ensure reproducibility of our analysis, allocation of raw data files to study participants is provided in Table S1.

Research involving human participants, their data, or biological material

Policy information about studies with human participants or human data. See also policy information about sex, gender (identity/presentation), and sexual orientation and race, ethnicity and racism.

Reporting on sex and gender	Self-reported sex was considered during recruitment and as metadata variable in principal component analysis. The linear mixed model used to identify differences in protein abundance was extended to include sex and all two-way interactions as fixed factors.
Reporting on race, ethnicity, or other socially relevant groupings	N/A
Population characteristics	Detailed participant characteristics are provided in Supplementary table S1.
Recruitment	Study eligibility was assessed for 28 healthy recreationally active runners aged between 18 and 35. To ensure complication-free execution of the high-intensity interval exercise on the treadmill, participants required a weekly running volume of 2-5 hours and a body mass index < 30. Any previous medical history of muscle disorders, cardiac or kidney diseases as well as regular intake of medication or nutritional supplements were treated as exclusion criteria. For female participants, breast-feeding or an ongoing pregnancy were also treated as exclusion criteria. Of the 28 subjects assessed for eligibility, two were considered ineligible due to acute infections. The remaining 26 participants provided written informed consent and were enrolled in the study. After baseline testing two further participants dropped out due to orthopedic problems while running (Achilles tendon injuries). For one participant, biomaterial did not suffice to run analyses, which resulted in a total of 23 participants.

Ethics oversight	Prior to enrollment of the first participants the study received ethical approval by the local ethics committee of the German Sport University Cologne, which works according to the World Medical Association's Declaration of Helsinki. The study meets the National Institutes of Health definition of a clinical trial and was prospectively registered in the German Clinical Trials Register (DRKS00017686).

Note that full information on the approval of the study protocol must also be provided in the manuscript.

Field-specific reporting

Please select the one below that is the best fit for your research. If you are not sure, read the appropriate sections before making your selection.

[x] Life sciences [ ] Behavioural & social sciences [ ] Ecological, evolutionary & environmental sciences

For a reference copy of the document with all sections, see nature.com/documents/nr-reporting-summary-flat.pdf

Life sciences study design

All studies must disclose on these points even when the disclosure is negative.

Sample size	Sample size was chosen based on previous studies investigating biological exercise effects using omics approaches in humans. To ensure analytic quality of our study setup we chose a conservative approach by recruiting > 20 participants for a randomized crossover study with repeated baselines. The high homogeneity of our study population, the low intra-individual variability of our analytic outcomes and the large amount of time, condition, and time × condition interaction effects indicate that our sample size was large enough to investigate the impact of acute exercise on immune cell numbers and protein abundance.
Data exclusions	Due to technical problems with the flow cytometer data from one participant could not be analyzed, resulting in a total n of 22. Additionally, for one sample biomaterial did not suffice to run the analysis, resulting in an n of 21 for 1h after MICE.
Replication	The applied crossover design enabled us to calculate intra-individual variability between the two baselines of each participant. For the proteomics analysis, the overall mean difference between the two baselines amounted to 0.13 ± 0.75 % for females and 0.06 ± 0.59 % for males (Figure 2B). Similarly, flow cytometry results of did not show significant differences between the two baselines (Figure S1B). These results underline the reproducibility of our analytic approach.
Randomization	Participants were randomized into one of two exercise intervention sequences after baseline testing: HIIE-MICE or MICE-HIIE. Following the minimization procedure by Pocock and Simon, randomization was performed via concealed allocation (1:1) using the software Randomization in Treatment Arms (RITA; Evidat, Lübeck, Germany). Age, BMI, and cardiorespiratory fitness (VO2peak) were used as stratification factors.
Blinding	The investigators performing flow cytometry-based immune cell phenotyping and the investigators performing LC-MS/MS-based untargeted proteomics were blinded to the participants' group allocation.

Reporting for specific materials, systems and methods

We require information from authors about some types of materials, experimental systems and methods used in many studies. Here, indicate whether each material, system or method listed is relevant to your study. If you are not sure if a list item applies to your research, read the appropriate section before selecting a response.

Materials & experimental systems

n/a	Involved in the study	
[ ]	[x]	Antibodies
[x]	[ ]	Eukaryotic cell lines
[x]	[ ]	Palaeontology and archaeology
[x]	[ ]	Animals and other organisms
[x]	[ ]	Clinical data
[x]	[ ]	Dual use research of concern
[x]	[ ]	Plants

Methods

n/a	Involved in the study	
[x]	[ ]	ChIP-seq
[ ]	[x]	Flow cytometry
[x]	[ ]	MRI-based neuroimaging

Antibodies

Antibodies used	anti-CD3 (BD Biosciences (cat. nr. 564001), BUV395, clone: SK7), Dilution factor: 1:400<br>anti-CD4 (BD Biosciences (cat. nr. 345770), PerCP, clone: SK3), Dilution factor: 1:80<br>anti-CD8 (BD Biosciences (cat. nr. 747097), BV750, clone: SK1), Dilution factor: 1:800<br>anti-CD16 (BD Biosciences (cat. nr. 557744), PE-Cy7, clone: 3G8), Dilution factor: 1:800<br>anti-CD25 (BD Biosciences (cat. nr. 742011), BUV805, clone: M-A251), Dilution factor: 1:400<br>anti-CD56 (BD Biosciences (cat. nr. 612928), BUV563, clone: NCAM16.2), Dilution factor: 1:400<br>anti-CD20 (BD Biosciences (cat. nr. 340908), APC, clone: L27), Dilution factor: 1:80<br>anti-CD19 (BD Biosciences (cat. nr. 566103), BV480, clone: SJ25C1), Dilution factor: 1:800

anti-Foxp3 (BD Biosciences (cat. nr. 560852), PE, clone: 259D/C7), Dilution factor: 1:200

Validation

Each antibody used for flow cytometry has been validated by the manufacturer. A link to each antibody´s product sheet including citations of publications using this product is included.
anti-CD3: https://www.bdbiosciences.com/content/dam/bdb/product_assets/product_pdf/singcolorpureantibody/buv395/pdf_0/564001.pdf
anti-CD4: https://www.bdbiosciences.com/content/dam/bdb/product_assets/product_pdf/singcolorpureantibody/bv605/pdf_0/23-5025.pdf
anti-CD8: https://www.bdbiosciences.com/content/dam/bdb/product_assets/product_pdf/singcolorpureantibody/bv750/pdf_0/747097.pdf
anti-CD16: https://www.bdbiosciences.com/content/dam/bdb/product_assets/product_pdf/singcolorpureantibody/pe-cy7/pdf_0/557744.pdf
anti-CD25: https://www.bdbiosciences.com/content/dam/bdb/product_assets/product_pdf/singcolorpureantibody/buv805/pdf_0/742011.pdf
anti-CD56: https://www.bdbiosciences.com/content/dam/bdb/product_assets/product_pdf/singcolorpureantibody/buv563/pdf_0/612928.pdf
anti-CD20: https://www.bdbiosciences.com/content/dam/bdb/product_assets/product_pdf/singcolorpureantibody/percp-cy55/pdf_0/23-5067.pdf
anti-CD19: https://www.bdbiosciences.com/content/dam/bdb/product_assets/product_pdf/singcolorpureantibody/bv480/pdf_0/566103.pdf
anti-Foxp3: https://www.bdbiosciences.com/content/dam/bdb/product_assets/product_pdf/singcolorpureantibody/pe/pdf_1/560852.pdf

Plants

Seed stocks

Report on the source of all seed stocks or other plant material used. If applicable, state the seed stock centre and catalogue number. If plant specimens were collected from the field, describe the collection location, date and sampling procedures.

Novel plant genotypes

Describe the methods by which all novel plant genotypes were produced. This includes those generated by transgenic approaches, gene editing, chemical/radiation-based mutagenesis and hybridization. For transgenic lines, describe the transformation method, the number of independent lines analyzed and the generation upon which experiments were performed. For gene-edited lines, describe the editor used, the endogenous sequence targeted for editing, the targeting guide RNA sequence (if applicable) and how the editor was applied.

Authentication

Describe any authentication procedures for each seed stock used or novel genotype generated. Describe any experiments used to assess the effect of a mutation and, where applicable, how potential secondary effects (e.g. second site T-DNA insertions, mosiacism, off-target gene editing) were examined.

Flow Cytometry

Plots

Confirm that:

☒ The axis labels state the marker and fluorochrome used (e.g. CD4-FITC).

☒ The axis scales are clearly visible. Include numbers along axes only for bottom left plot of group (a 'group' is an analysis of identical markers).

☒ All plots are contour plots with outliers or pseudocolor plots.

☒ A numerical value for number of cells or percentage (with statistics) is provided.

Methodology

Sample preparation

Cryopreserved PBMCs from all participants were gently thawed in a water bath at 37 °C with a mean recovery of 81.28 % viable cells assessed with the Zombie NIR™ Fixable Viability Kit (BioLegend, San Diego, CA, USA). After incubating 1 × 106 PBMCs in 2.5 µg Fc block for 10 min at room temperature, cells were stained with anti-CD3 (BUV395, clone SK7), anti-CD4 (PerCP, clone SK3), anti-CD8 (BV750, clone SK1), anti-CD16 (PE-Cy7, clone 3G8), anti-CD25 (BUV805, clone M-A251), anti-CD56 (BUV563, clone NCAM16.2), anti-CD20 (APC, clone L27), and anti-CD19 (BV480, clone SJ25C1) antibodies (all from BD Biosciences, NJ, USA). In brief, cells were incubated in the dark with a master mix containing Brilliant Stain buffer (BD Biosciences) and antibodies against surface antigens for 30 min at 4°C. After washing with FACS buffer, the BD Pharmingen™ Transcription Factor Buffer Set was used, and cells were fixed for 40 min at 4 °C in the dark. Thereafter, intracellular staining was done by incubating cells with an anti-Foxp3 antibody (PE, clone 259D/C7) for 45 min at 4 °C in the dark. After washing, cells were resuspended in FACS buffer and acquired on the flow cytometer within 2 hours after finishing the staining protocol.

Instrument

Flow cytometry analysis was performed using a Cytek® Aurora full spectrum flow cytometer equipped with 5 lasers (Cytek Biosciences, California, USA).

Software

Flow cytometry analysis was performed using SpectroFlo® software (Cytek Biosciences, California, USA). Gating of flow cytometry results was performed using FlowJo™ 10.10.0 (BD Biosciences, NJ, USA).

Cell population abundance

N/A

Gating strategy

Lymphocytes were gated using forward scatter (FSC) area and sideward scatter (SSC) area. Single cells were gated using FSC height and area as well as SSC height and area. Vital cells were gated as Zombie NIR™ negative events using the Zombie NIR™ Fixable Viability Kit (BioLegend, San Diego, CA, USA). B cells were phenotyped as CD3-CD56-CD19+CD20+, natural killer T (NKT) cells as CD3+CD56+, natural killer (NK) cells either as CD56brightCD16- (NKbright) or CD56dimCD16+ (NKdim), T cells as CD3+CD56-, T helper cells as CD4+CD8-, cytotoxic T cells as CD4-CD8+, and regulatory T cells (Tregs) as CD4+CD25+Foxp3+.

☒ Tick this box to confirm that a figure exemplifying the gating strategy is provided in the Supplementary Information.
